# Differences Between Young and Older Adults in Working Memory and Performance on the Test of Basic Auditory Capabilities^†^

**DOI:** 10.3389/fpsyg.2021.804891

**Published:** 2022-01-12

**Authors:** Larry E. Humes, Gary R. Kidd, Jennifer J. Lentz

**Affiliations:** Department of Speech, Language, and Hearing Sciences, Indiana University, Bloomington, IN, United States

**Keywords:** aging, auditory perception, cognition, hearing loss, auditory discrimination and identification

## Abstract

The Test of Basic Auditory Capabilities (TBAC) is a battery of auditory-discrimination tasks and speech-identification tasks that has been normed on several hundred young normal-hearing adults. Previous research with the TBAC suggested that cognitive function may impact the performance of older adults. Here, we examined differences in performance on several TBAC tasks between a group of 34 young adults with a mean age of 22.5 years (SD = 3.1 years) and a group of 115 older adults with a mean age of 69.2 years (SD = 6.2 years) recruited from the local community. Performance of the young adults was consistent with prior norms for this age group. Not surprisingly, the two groups differed significantly in hearing loss and working memory with the older adults having more hearing loss and poorer working memory than the young adults. The two age groups also differed significantly in performance on six of the nine measures extracted from the TBAC (eight test scores and one average test score) with the older adults consistently performing worse than the young adults. However, when these age-group comparisons were repeated with working memory and hearing loss as covariates, the groups differed in performance on only one of the nine auditory measures from the TBAC. For eight of the nine TBAC measures, working memory was a significant covariate and hearing loss never emerged as a significant factor. Thus, the age-group deficits observed initially on the TBAC most often appeared to be mediated by age-related differences in working memory rather than deficits in auditory processing. The results of these analyses of age-group differences were supported further by linear-regression analyses with each of the 9 TBAC scores serving as the dependent measure and age, hearing loss, and working memory as the predictors. Regression analyses were conducted for the full set of 149 adults and for just the 115 older adults. Working memory again emerged as the predominant factor impacting TBAC performance. It is concluded that working memory should be considered when comparing the performance of young and older adults on auditory tasks, including the TBAC.

## Introduction

The World Health Organization (2021) estimated that there are 162 million older adults worldwide with disabling age-related hearing loss. World Health Organization (2021) estimates the prevalence of such audiometrically defined disabling hearing loss to be 25% for those over 60, increasing from 15.4% globally among people aged in their 60s to 58.2% globally for those over 90 years old. Audiometric hearing loss for pure tones, however, captures just one aspect of auditory function in adults that can lead to limitations in activity and restrictions on participation in society, according to the widely applied World Health Organization (2001) model of healthy function. Other measures of auditory function beyond the audiogram may have implications for healthy living as well.

Humes et al. (2012), based on a review of 165 articles published in the peer-reviewed literature between 1988 and 2012, found evidence for declines in various measures of auditory abilities with advancing age. The bulk of the research over the review period in Humes et al. (2012) was on auditory temporal processing. Importantly, Humes et al. (2012) noted that it was difficult to ascertain whether the observed declines in auditory abilities with age reflected deficits in higher-level auditory processing or were driven by concomitant declines in hearing threshold, cognitive function, or both. A recent review by Gallun and Best (2020) provides support for the existence of age-related declines in auditory processing but also notes concerns about possible peripheral and cognitive confounds.

The Test of Basic Auditory Capabilities (TBAC) was developed by Watson et al. (1982a,b; see Watson, 1987) as an easy-to-administer battery of auditory processing that tapped several auditory abilities. The original TBAC included three single-tone discrimination tests, three tests of temporal pattern discrimination, and two tests using syllables, one assessing temporal-order discrimination and the other syllable identification in noise. Several subsequent studies have employed versions of the TBAC with large numbers of young normal-hearing (YNH) adults (Watson and Miller, 1993; Surprenant and Watson, 2001; Kidd et al., 2007). The TBAC has also been found to be reliable in YNH listeners (Kidd et al., 2007) and in older adults with hearing impairment (OHI) of varying degrees (Christopherson and Humes, 1992).

The TBAC has been used to compare the auditory-processing performance of YNH and OHI listeners in some prior studies as well. Humes and Christopherson (1991), for example, compared the performance of 23 older adults, 65–86 years of age, to that of YNH adults listening either in quiet (*N* = 10; 19–36 years) or in a background of noise designed to simulate the average hearing loss of the OHI listeners (*N* = 12; 20–31 years). Significant deficits were observed in the performance of the OHI group compared to both YNH groups on 4 of the 8 TBAC tests: frequency discrimination and three measures of temporal processing (an embedded test-tone duration-discrimination task and two temporal-order discrimination tasks: one using pure tones and the other using syllables). In addition, although hearing loss was the primary factor affecting speech-identification, some TBAC measures (notably, frequency discrimination) accounted for small but significant improvements in predictions of speech-identification performance.

Humes and Christopherson (1991) did not obtain measures of cognitive function in their study and subsequent work by Watson and Miller (1993) showed a link between cognitive function and TBAC performance in a large group of YNH listeners. As noted in Humes (1996), this led to replication of the Humes and Christopherson (1991) study, but this time using YNH and OHI groups matched for hearing loss and cognitive function. When doing so, no differences in TBAC performance were observed between young and older adults. This suggested that the prior “age group” difference may have been driven by concomitant age-group differences in hearing loss, cognitive function, or both.

In another study with OHI listeners, Humes et al. (1994) examined individual differences in TBAC performance among a group of 50 older adults ranging in age from 63 to 83 years and having varying degrees of hearing loss. In addition to the TBAC, cognitive function was assessed with the Wechsler Adult Intelligence Scale-Revised (WAIS-R; Wechsler, 1981) and the Wechsler Memory Scale-Revised (Wechsler, 1983). The primary focus of the investigation by Humes et al. (1994) was on the association of both the TBAC and the cognitive measures with unaided speech-recognition performance. Although not the focus, moderate correlations were evident between performance on the TBAC and performance on the cognitive measures.

More recently, Humes et al. (2013b) measured auditory performance in a group of 98 older adults making use of 27 different stimulus conditions to measure seven main auditory psychophysical abilities. In addition, the TBAC was employed, but the results were only presented for the mean performance on 6 of the 8 TBAC tests; the six discrimination tasks making use of tonal stimuli. The two TBAC tests making use of syllables were omitted because the focus of the study by Humes et al. (2013b) was on the identification of factors underlying individual differences in aided speech perception and the authors felt it was inappropriate to use speech-based tests to predict performance on other speech-based tests. Given the large number of other auditory measures in Humes et al. (2013b), only the mean TBAC performance for the six tonal tests, referred to as TBAC6, was considered. This 6-test mean TBAC score was found to be reliable in a group of 31 older adults with a test-retest correlation of *r* = 0.76 but mean retest scores were slightly (79.1 vs 75.4% correct) and significantly (*p* < 0.001) higher than the test scores. When comparing the mean performance of the 98 older adults to that of a normative group of 27 YNH listeners, the older group had significantly (*p* < 0.01) lower TBAC6 scores (82.9 vs. 76.1% correct). Because performance on the TBAC was not well represented in the principal-components solution for the large set of auditory psychophysical measures in that study, it was dropped by Humes et al. (2013b) from subsequent regression analyses.

In the present study, we looked more carefully at the performance of older adults on the TBAC using the data collected originally by Humes et al. (2013b). Rather than only averaging across the six TBAC tests making use of tonal stimuli, performance on each of eight TBAC tests was examined separately, as in earlier studies with the TBAC. Because measures of hearing loss and cognitive function were also available from most of the study participants, we also examined the relative contributions of these factors to TBAC performance. Given the prior observations of differences in performance between YNH and OHI listeners on many of the TBAC tests, we addressed whether such age-group differences remained after statistically controlling for differences in hearing loss and cognitive function between the two age groups.

## Materials and Methods

### Participants

There were 115 adults in the OHI group and 34 adults in the YNH group for these analyses. Both groups are larger than those in Humes et al. (2013b), as only those with complete data across the full set of psychophysical, cognitive, and speech-recognition measures were included in those prior analyses. Here, participants only needed to complete the tests used to determine study eligibility, including an audiogram, three brief cognitive tests and the TBAC. Because all these measures were obtained in the early part of the lengthy data-collection process, data were available for these measures from larger samples of older and younger adults than in Humes et al. (2013b).

The group of older adults included 56 women (48.7%) and 59 men (51.3%), with a mean age of 69.2 years (SD = 6.2 years). The group of young adults included 26 women (76.5%) and eight men (23.5%), with a mean age of 22.5 years (SD = 3.1 years). None of the participants were current hearing-aid users and 90% of the older adults had never worn hearing aids. All subjects had no evidence of middle-ear pathology (air-bone gaps < 10 dB and normal tympanograms bilaterally), no signs of dementia (Mini Mental Status Exam, MMSE, > 25; Folstein et al., 1975), and had English as his or her native language. Older subjects were recruited primarily via newspaper ads in the local paper and younger subjects by flyers and university online postings.

The study protocol was approved by the Indiana University-Bloomington Institutional Review Board prior to data collection. All subjects signed informed consent forms for the study and the use of their de-identified data for research purposes. All subjects were paid for their participation.

**FIGURE 1:**
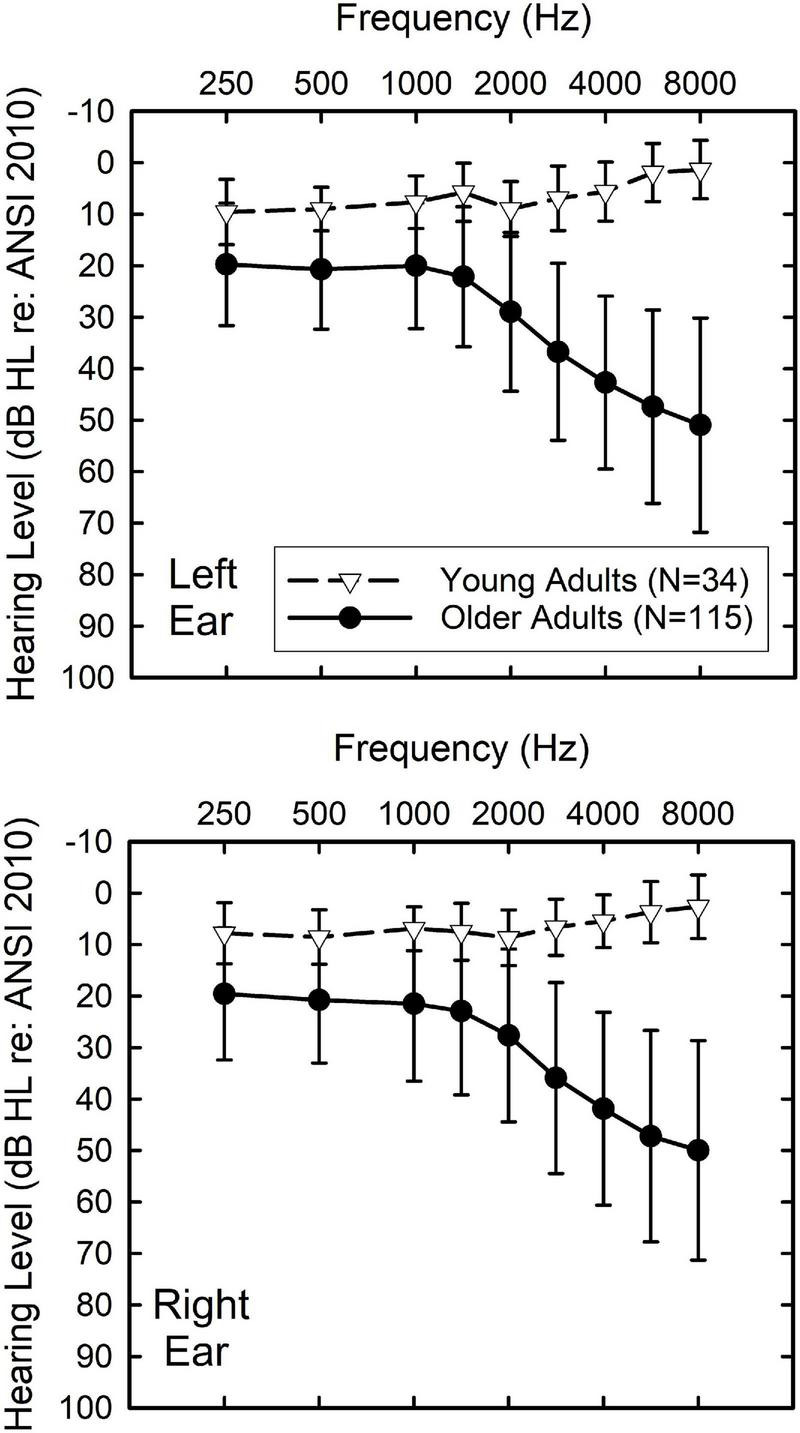
Means and standard deviations for the air-conduction pure-tone thresholds from the audiogram for young (triangles) and older (circles) adults and the left (top) and right (bottom) ear.

For the older adults, the primary audiometric inclusion criterion was bilaterally symmetrical hearing with the threshold at 4,000 Hz ≤ 60 dB HL (ANSI, 2004) in at least one ear. This maximum hearing loss at 4,000 Hz was established to ensure that the spectrally shaped speech stimuli used in other portions of the study would be fully audible through 4,000 Hz. For the young adults, hearing thresholds were ≤25 dB HL from 250 through 8,000 Hz in both ears. The means and standard deviations for the air-conduction hearing thresholds of each ear are shown for each group in Figure 1. When controlling for the effects of hearing loss on TBAC performance in the analyses, the average threshold for 500, 1,000, 2,000, and 4,000 Hz, PTA4, will be used. As noted below, the TBAC is presented diotically. As a result, the better-ear PTA4 was used in the analyses below when controlling for hearing loss. For the older adults, the mean better-ear PTA4 = 25.3 dB HL (SD = 11.1 dB HL), and, for the young adults, the mean better-ear PTA4 = 6.5 dB HL (SD = 3.4 dB HL) which was a significant difference [*t* (147) = 9.7, *p* < 0.001] with a very large effect size (Cohen’s *d* = 1.9; Cohen, 1988). Although this difference is significant, 59% of the older adults had normal hearing, as defined by better-ear PTA4 ≤ 25 dB HL, and 42% when defined as better-ear PTA4 ≤ 20 dB HL.

### Equipment and Materials

All testing was conducted with participants seated in a sound-attenuating room. All TBAC stimuli were played through a 16-bit high-quality sound card (Digital Audio Labs Card Deluxe) with a sampling rate of 44,100 Hz. The output was fed into Etymotic Research ER-3A insert earphones. TBAC stimuli were presented diotically at a level of 85 dB SPL as measured in a 2-cm^3^ coupler. The relatively high presentation level was used to further minimize the impact of elevated hearing thresholds on TBAC performance for the older adults.

#### The Test of Basic Auditory Capabilities

The version of the TBAC used here was the TBAC-4, obtained from Communication Disorders Technology (CDT), Inc. The test battery, equivalent to that used in the earlier work with OHI listeners, includes six tests of auditory discrimination using tones, and two tests using speech sounds. The eight tests are briefly described below. For additional details see Kidd et al. (2007) and the TBAC information available on the CDT web site.^1^

Trials in each of the tests, except for the last test (syllable identification), were structured in a modified two-alternative forced-choice (2AFC) format in which a standard stimulus was followed by two test stimuli, one of which was different from the standard. The listeners used a computer keyboard to indicate which test stimulus was different from the standard. Trials were arranged in groups of six, and the level of difficulty was systematically increased from trial to trial, within each group, in logarithmic steps. For seven of the eight TBAC tests, eight levels of difficulty were tested over 72 trials, presenting the six easiest levels in the first 36 trials, followed by an increase in difficulty of two log steps for trials 37–72. For the penultimate test, the temporal-order task using syllables as stimuli, only five levels of difficulty were included with a total of 48 trials.

The TBAC administered here was comprised of eight tests. Each test is described briefly here.

*Single-tone frequency discrimination (dF)* for which the standard was a 1,000-Hz 250-ms tone and frequency increments were used.

*Single-tone intensity discrimination (dI)* for which the standard was a 1,000-Hz 250-ms tone and intensity increments were used.

*Single-tone duration discrimination (dT)* for which the standard was a 1,000-Hz 100-ms tone and duration increments were used.

*Pulse-train discrimination (dPT;* rhythm) with the standard consisting of six 20-ms pulses (1,000-Hz tone) arranged in three pairs, with a 40-ms pause within a pair and a 120-ms pause between pairs. The “different” sequence included an increase in the duration within a pair with a corresponding decrease in the duration between pairs, altering the rhythm of the sequence while keeping the total duration constant.

*Embedded tone detection (dETT)* with the standard consisting of a sequence of eight tones of differing frequency with a temporal gap (ranging from 10 to 200 ms) in the middle of the sequence. The “different” sequence had a tone (also ranging from 10 to 200 ms in duration) filling the temporal gap in the middle position. A different sequence of frequencies (ranging from 300 to 3,000 Hz) was presented on each trial. The duration of the middle gap or tone was varied to manipulate task difficulty.

*Temporal-order discrimination for tones (dTOpt)* for which the standard was a four-tone pattern consisting of two equal-duration tones (550 and 710 Hz) preceded and followed by a 100-ms 625-Hz tone. The middle tones were presented in reverse order in the “different” interval. The duration of the tones varied from 20 to 200 ms in equal-log steps. Shrivastav et al. (2008) found that the resulting variations in both rate of presentation and tone duration impact the performance of OHI listeners on this task.

*Temporal-order discrimination for syllables (dTOsyl)* is similar to the preceding test, but with consonant-vowel (CV) syllables comprising the sequence instead of tones. The task is to discriminate /fa/-/ta/-/ka/-/pa/ from /fa/-ka/-/ta/-/pa/. The duration of the syllables was varied (by reducing the vowel duration) from 250 to 75 ms in five steps.

*Syllable identification (SylID)* was a test of the recognition of nonsense CVC syllables in broadband noise. A 3AFC paradigm was used, with foils created by altering the vowel or one of the consonants. Five speech-to-noise ratios (SNRs) were used with decreasing SNRs within each set of five trials. A set of 100 stimuli was presented twice in separate blocks, with a different random order for each block.

#### Working Memory Tests

Three tests from a Matlab-based working memory test battery developed by Lewandowsky et al. (2010) were administered. For all tests, there were no time constraints on the recall task at the end of each trial and no feedback was provided. Each test took approximately 10 mins to complete. All testing took place with the participant comfortably seated in front of a computer monitor and keyboard inside a sound-attenuating booth. Procedural modifications to accommodate the older participants were implemented by Humes et al. (2013b) and are noted again here.

#### Memory Updating

At the start of each trial, subjects were presented with a sequence of three to five digits. Each digit was surrounded by a square to mark its position on the screen. After all digits were presented, the squares remained on the screen and a different sequence of arithmetic operations (addition or subtraction, with numbers ranging from +7 to -7) appeared in each of the squares, one at a time. The subject’s task was to remember the digits that appeared in each square and then perform the sequence of arithmetic operations presented in each of the squares. The subject was asked to indicate (using the keyboard) the final resulting value in each square after a sequence of two to six sequential arithmetic operations. Consider the following example for a set size of 3. Three digits, 2 4 1, appear on the screen, one in each square. The digits are then replaced by +1 -2 +5 and these mathematical operations are applied to the digits retained in memory such that the new 3-digit sequence in memory is 3 2 6. Next, another set of three mathematical operations appear in the three squares on the screen: +2 +3-1. The new sequence in memory is now 5 5 5. For two sequential operations, the task ends, and the subject would enter 5 5 5 as the response. Otherwise, this process continues for up to a total of six sequential operations before the total from memory is requested as the response. The test consisted of 15 trials with a randomly generated sequence of set size (3–5 co-occurring series of operations) and number of operations (2–6) on each trial.

Because this test was challenging for older adults, some adjustments were made to the procedures to ensure that the task was well understood, and to make it a bit less challenging. The number of practice trials was increased from two (the default) to four, and the time between items (to be added or subtracted) was increased from 250 to 500 ms. The first two practice trials used a 3-s inter-item time to allow the experimenter to explain the required operations during the trial. Also, the default instructions were supplemented with a verbal explanation of the task that included a subject-paced simulated trial using cue cards to present the stimuli.

#### Sentence Span

The “easy” version of the sentence-span task was used for this study. In this task, subjects were presented with an alternating sequence of simple sentences (3–6 words in length) and single letters on the computer screen. Subjects judged whether the sentence was true or false on each presentation, with 4 s allowed for responding. The letters required no response. After from four to eight sentence/letter presentations, subjects were asked to recall the letters in the order they were presented. The test consisted of 15 trials (after three practice trials) with three instances of each number of sentence/letter presentations.

#### Spatial Short-Term Memory

This test assessed a subject’s ability to recall the location of dots (filled circles) in a 10 × 10 grid. On each trial, an empty grid was presented and then a sequence of dots appeared in the grid. Each dot remained on the screen for approximately 1 s before it was removed, and the next dot appeared. From two to six dots were presented on each trial. After all the dots had been presented (and removed), the subject was asked to indicate the relative position of the dots by touching (or pointing and clicking with a computer mouse) the cells within the grid. This test consisted of 30 trials (6 at each set size).

## Results

### Reliability

Of the 115 older adults who completed the TBAC, 29 (25%) repeated the TBAC after completion of all other measures in the larger psychophysical study (Humes et al., 2013b) to provide an assessment of TBAC reliability. As noted, the test-retest data were only reported for the 6-test average of the TBAC, TBAC6, in Humes et al. (2013b). Table 1 summarizes the results from the test-retest analyses for all 8 TBAC tests and the TBAC6 average. Performance on only two TBAC measures, the dF test and the TBAC6 average score, showed significant changes from test to retest with both showing a 4–5% point improvement on retest. Six of the nine test-retest correlations in Table 1 are significant (*p* < 0.05, adjusted for multiple comparisons), SylID being the only test with poor test-retest correlation (*r* = −0.03). Of the remaining eight test-retest correlations in Table 1, all are moderate in strength and six of the eight are significant. Not surprisingly, the strongest test-retest correlation was observed for the score based on the most trials, the TBAC6 average score.

**TABLE 1:** Test and retest means (M) and standard deviations (SD) for 29 of the 115 older adults.

TBAC Test	Test M%	Test SD%	Retest M%	Retest SD%	*p*_t_ *	*r*	*p*_r_ *
dF	**76.3**	**9.6**	**81.5**	**7.2**	**0.002**	**0.58**	**< 0.001**
dI	84.0	10.4	88.0	8.6	0.026	**0.55**	**0.002**
dT	74.0	10.2	78.2	11.6	0.006	**0.68**	**< 0.001**
dPT	80.7	12.6	85.6	8.4	0.012	0.45	0.015
dETT	71.4	10.8	74.6	7.8	0.063	**0.52**	**0.004**
dTOpt	67.3	9.7	66.6	9.5	0.503	**0.55**	**0.002**
dTOsyl	55.1	10.1	56.3	11.5	0.553	0.45	0.015
SylID	53.4	10.5	56.8	6.7	0.164	–0.03	0.860
TBAC6	**75.6**	**7.5**	**79.1**	**5.6**	**< 0.001**	**0.76**	**< 0.001**

Test-retest correlations (r), and their significance (p_r_), are also shown. Significance of differences in means between test and retest and of the correlations is also shown (p_t_). Entries in bold font indicate either significant differences between means (p_t_) or correlations (p_r_). *p values adjusted for multiple comparisons with criterion p < 0.05/9 or p < 0.0055.

We also explored whether the reliability would be further enhanced by averaging all seven of the auditory discrimination measures, but the test-retest correlation for this 7-test average score decreased slightly to *r* = 0.72 (*p* < 0.001) compared to the 6-test average (*r* = 0.76, *p* < 0.001). Finally, we generated a 4-test average for the four pure-tone discrimination tasks which also had the four highest test-retest correlation in Table 1: dF, dI, dT, and dTOpt. The test-retest correlation for this TBAC4 average score was *r* = 0.71 (*p* < 0.001). In summary, individual test scores from the TBAC show moderate reliability among older adults and the reliability is enhanced when various average scores are used, with the TBAC6 average proving to be the most reliable, although the differences in *r* values among the various TBAC averages are not significant (*p* > 0.1).

The reliability of the working-memory tests had been established in older adults previously by Humes et al. (2013b). For the three working-memory tests, Humes et al. (2013b) reported that there were no significant changes in mean performance from test to retest and the test-retest correlations were *r* = 0.69, 0.83, and 0.83 for the spatial STM, sentence span, and memory updating tests, respectively.

### Age-Group Differences in Working Memory

As noted, the YNH and OHI groups not only differed significantly in age but also in average hearing loss (better-ear PTA4, Figure 1). Age-group differences were also expected for the measures of working memory (e.g., Salthouse, 2010). This was confirmed here for the data from the 34 YNH and 115 OHI participants. The means (and standard deviations) for the percent-correct scores from the young adults were 74.7 (11.0), 72.2 (11.7), and 84.8 (5.0) for memory updating, sentence span, and spatial STM tasks, respectively. For the older adults, the means (and standard deviations) were 49.8 (20.5), 52.6 (16.5), 72.7 (6.8). Independent-sample *t*-tests were significant for all three working-memory tests [all *t* (147) > 6.4, *p* < 0.001]. This was also true for a single principal-component score (accounting for 73.6% of the variance) derived from principal-component analysis of the three working-memory scores. The means (and standard deviations) for the working-memory principal component (PCwm) were 1.13 (0.45) and -0.36 (0.87) for the young and older adults, respectively. The independent-samples *t*-test resulted in *t* (147) = 9.3, *p* < 0.001.

### Age-Group Differences on the Test of Basic Auditory Capabilities

**FIGURE 2:**
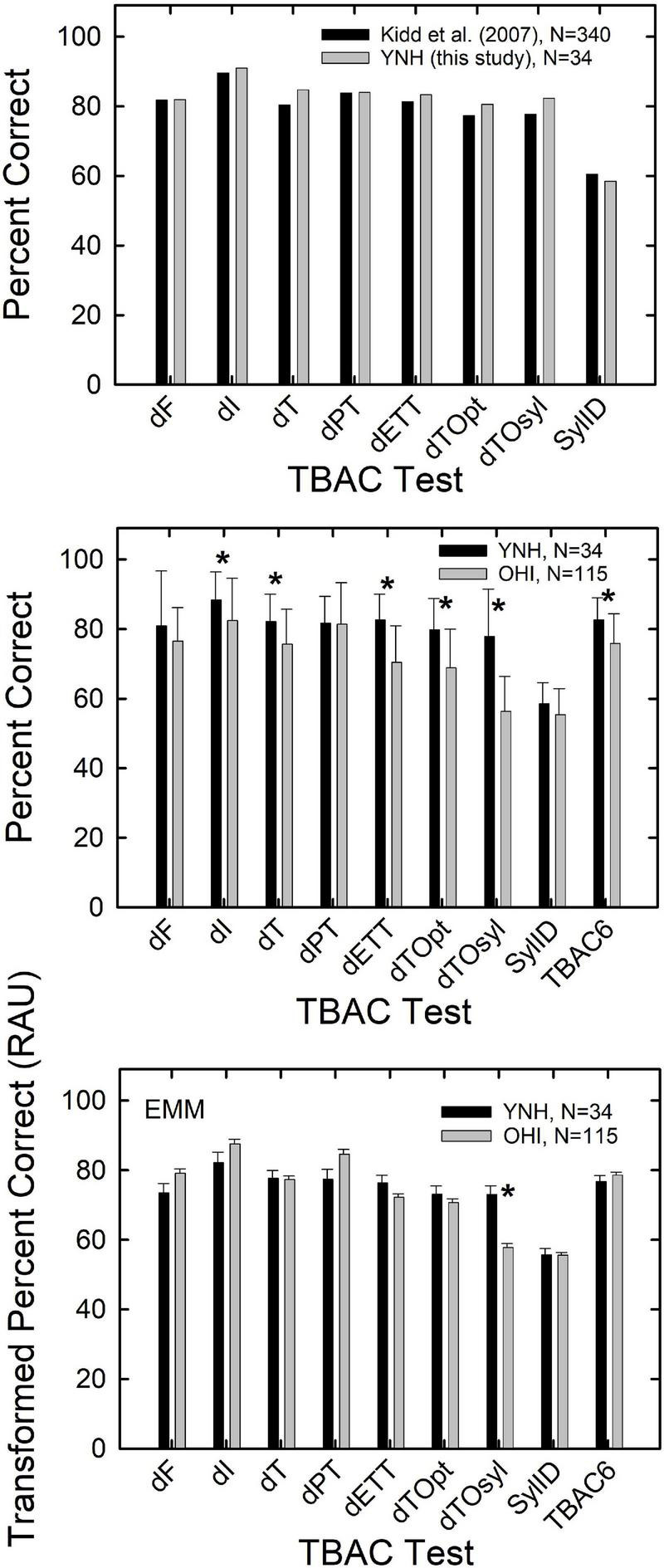
The top panel compares the mean percent-correct performance for young normal-hearing (YNH) adults in this study (*N* = 34; gray bars) to the corresponding mean normative values from Kidd et al. (2007; *N* = 340; black bars). The middle panel shows the means and standard deviations for the percent-correct scores on the TBAC for the 34 YNH (black bars) and 115 older hearing-impaired (OHI; gray bars) adults in this study. The bottom panel provides the estimated marginal means (EMM, controlling for PTA4 and working memory) and standard errors for the YNH (black bars) and OHI (gray bars) groups in this study. The asterisks in the lower two panels mark significant effects (adjusted *p* < 0.0055) of subject group.

Figure 2 shows the TBAC scores for the YNH and OHI groups compared in various ways. In the top panel, the mean scores from the 34 YNH adults in these analyses (gray bars) are compared to the largest set of normative data obtained by Kidd et al. (2007) from 340 YNH adults (black bars). No statistical analyses were performed on the data in the top panel. Rather, the similarity of the means for both groups of YNH listeners is just offered as evidence that the performance of our group of 34 YNH adults on the TBAC appears to be representative or typical for YNH adults generally for this battery of tests.

The middle panel of Figure 2 shows the means and standard deviations for the YNH and OHI groups in these analyses. Data are shown for each of the eight TBAC tests as well as the mean percent-correct score for the six tonal auditory-discrimination tasks (TBAC6). The TBAC percent-correct scores were transformed to rationalized arcsine units (RAU; Studebaker, 1985) to stabilize the error variance prior to analysis of variance (ANOVA) for the group effects. With a Bonferroni-adjusted *p* value of 0.0055 (0.05/9), 6 of the 9 group differences were significant with the young adults outperforming the older adults in each case [all *F* (1,147) > 7.96, *p* < 0.005]. For the six significant group effects, all eta-squared effect sizes were >0.05 indicating that all effect sizes were at least medium effects (Cohen, 1988). The three non-significant group differences were for the dF, dPT, and SylID TBAC tests.

As noted, the YNH and OHI groups not only differed significantly in age but also in average hearing loss (better-ear PTA4, Figure 1) and working memory. When analysis of covariance (ANCOVA) was performed on each of the nine TBAC measures, with covariates of better-ear PTA4 and overall working-memory performance (PCwm), significant group differences (*p* < 0.05 with Bonferroni adjustment to *p* < 0.0055) were observed for only one of the nine TBAC measures. This is shown in the bottom panel of Figure 2 which depicts the estimated marginal means (EMMs) for the rau-transformed TBAC scores after adjustment for the PTA4 and PCwm covariates. The lone significant difference between the YNH and OHI groups that remained after controlling for PTA4 and working memory was the temporal-order task with syllables [*F* (1,145) = 26.6, *p* < 0.001; eta squared = 0.15, large effect size). These analyses also found that the better-ear PTA4 covariate never had a significant effect on TBAC scores [all *F* (1,145) < 5.8, *p* > 0.02]. In contrast, the PCwm covariate was found to be significant in 8 of the 9 ANCOVAs [all *F* (1,145) > 12.2, *p* < 0.001] with medium to large effect sizes based on eta squared. The only TBAC measure that did not show a significant effect of working memory on performance was the syllable identification task [SylID; *F* (1,145) = 5.8, *p* > 0.01].

### Individual Differences in the Test of Basic Auditory Capabilities Scores

To further evaluate the effects of age, hearing loss, and working memory on TBAC performance, linear-regression analyses were also performed with each TBAC measure serving as the dependent variable. Table 2 summarizes the results for each of the nine regression analyses for the entire sample of 149 adults. The *F* values in the third column show that significant regression solutions emerged for all nine TBAC measures with the variance explained in each case shown by the r^2^ values in the preceding column. The rows in bold font in Table 2 mark those predictors found to be significant (*p* < 0.05, unadjusted). The final three columns in Table 2 provide the zero-order, partial, and part correlations for each predictor in each regression analysis. The partial correlation examines the association between an independent variable and a dependent variable after controlling for the influence of other variables on both the independent and dependent variable. The part or semi-partial correlation examines the association between the independent and dependent variable after controlling for the effects of the other variables on just the independent variable. Review of those last two columns of correlations reveals a very clear pattern. For 8 of the 9 TBAC measures, working-memory performance was either the only (seven times) or predominant (one time) significant predictor. The only exception was the temporal-order task for syllables, dTOsyl, for which working-memory was again significant but age was the predominant predictor. Recall that it was only this task that showed a significant difference between age groups in the previously presented ANCOVA. Thus, the linear-regression analyses of the individual data, summarized in Table 2, support the group analyses summarized previously in the bottom panel of Figure 2.

**TABLE 2:** Results of the linear-regression analyses for each the test of basic auditory capabilities (TBAC) score (in RAU) for 149 young and older adults.

TBAC Test	*r* ^2^	*F* (3,145)	Ind Var	Std Beta	*t*	*p*	*r*	Partial *r*	Part *r*
dF	0.162	9.32*	**PC WM**	**0.442**	**4.347**	**< 0.001**	**0.383**	**0.340**	**0.331**
			zAge	0.192	1.550	0.123	–0.194	0.128	0.118
			zPTA4	–0.133	–1.218	0.225	–0.224	–0.101	–0.093
dI	0.218	13.43*	**PC WM**	**0.455**	**4.635**	**< 0.001**	**0.455**	**0.359**	**0.340**
			zAge	0.117	0.971	0.333	–0.290	0.080	0.071
			zPTA4	–0.148	–1.405	0.162	–0.300	–0.116	–0.103
dT	0.235	14.87*	**PC WM**	**0.511**	**5.262**	**< 0.001**	**0.483**	**0.400**	**0.382**
			zAge	0.010	0.084	0.933	–0.299	0.007	0.006
			zPTA4	0.040	0.389	0.698	–0.216	0.032	0.028
dPT	0.087	4.60**	**PC WM**	**0.391**	**3.687**	**< 0.001**	**0.227**	**0.293**	**0.293**
			**zAge**	**0.261**	**2.011**	**0.046**	−**0.009**	**0.165**	**0.160**
			zPTA4	–0.015	–0.132	0.895	–0.031	–0.011	–0.010
dETT	0.355	26.62*	**PC WM**	**0.366**	**4.105**	**< 0.001**	**0.552**	**0.323**	**0.274**
			zAge	–0.155	–1.426	0.156	–0.512	–0.118	–0.095
			zPTA4	–0.161	–1.682	0.095	–0.460	–0.138	–0.112
dTOpt	0.312	21.90*	**PC WM**	**0.425**	**4.611**	**< 0.001**	**0.542**	**0.358**	**0.318**
			zAge	–0.122	–1.088	0.278	–0.453	–0.090	–0.075
			zPTA4	–0.070	–0.708	0.480	–0.376	–0.059	–0.049
dTOsyl	0.488	46.03*	**PC WM**	**0.241**	**3.035**	**0.003**	**0.581**	**0.244**	**0.180**
			**zAge**	−**0.519**	−**5.347**	**< 0.001**	−**0.675**	−**0.406**	−**0.318**
			zPTA4	0.005	0.061	0.951	–0.489	0.005	0.004
sylID	0.070	3.61***	**PC WM**	**0.219**	**2.042**	**0.043**	**0.259**	**0.167**	**0.164**
			zAge	–0.029	–0.219	0.827	–0.202	–0.018	–0.018
			zPTA4	–0.041	–0.357	0.722	–0.174	–0.030	–0.029
TBAC6	0.345	25.51*	**PC WM**	**0.575**	**6.398**	**< 0.001**	**0.582**	**0.469**	**0.430**
			zAge	0.076	0.696	0.488	–0.384	0.058	0.047
			zPTA4	–0.112	–1.170	0.244	–0.355	–0.097	–0.079

Bold font highlights those independent variables having significant (p < 0.05) standardized Beta coefficients in significant regression solution. Asterisks mark significant F values for the regression solution: *p < 0.001; **p < 0.01; ***p < 0.05.

A second set of linear-regression analyses was completed for all 9 TBAC measures as the dependent variable; this time for only the older adults. The age range, 60–88 years, was sufficient to expect some age-related changes in performance. The use of a narrower age range in such analyses can also provide stronger evidence of age-related effects on performance (e.g., Hofer and Sliwinski, 2001). Table 3 summarizes the results from the second set of regression analyses for the 115 older adults. The *F* values reveal that significant regression solutions were observed for all but the syllable-identification task (sylID). For the other 8 TBAC measures in Table 3, the partial and part correlations in the far-right columns indicate that working memory was always the predominant predictor, and in 6 of the 8 cases was the sole significant factor. For the two TBAC tests for which a second significant predictor emerged (dF, dETT), in both cases, the other predictor was the better-ear PTA4. In summary, among the older adults, ranging in age from 60 to 88 years, working memory was the sole or primary predictor of performance on the TBAC.

**TABLE 3:** Results of the linear-regression analyses for each TBAC score (in RAU) for 115 older adults only.

TBAC Test	r^2^	*F* (3,111)	Ind Var	Std Beta	*t*	*p*	*r*	Partial *r*	Part *r*
dF	0.257	12.77*	**PC WM**	**0.459**	**5.226**	**< 0.001**	**0.472**	**0.444**	**0.428**
			zAge	0.099	1.002	0.318	–0.173	0.095	0.082
			**zPTA4**	−**0.214**	−**2.253**	**0.026**	−**0.268**	−**0.209**	−**0.184**
dI	0.203	9.45*	**PC WM**	**0.354**	**3.897**	**< 0.001**	**0.417**	**0.347**	**0.330**
			zAge	–0.119	–1.161	0.248	–0.291	–0.110	–0.098
			zPTA4	–0.091	–0.921	0.359	–0.232	–0.087	–0.078
dT	0.200	9.24*	**PC WM**	**0.459**	**5.048**	**< 0.001**	**0.442**	**0.432**	**0.429**
			zAge	0.007	0.072	0.943	–0.122	0.007	0.006
			zPTA4	0.067	0.681	0.497	–0.033	0.064	0.058
dPT	0.097	3.97***	**PC WM**	**0.313**	**3.234**	**0.002**	**0.310**	**0.293**	**0.292**
			zAge	–0.012	–0.108	0.914	–0.108	–0.010	–0.010
			zPTA4	0.030	0.282	0.779	–0.047	0.027	0.025
dETT	0.184	8.36*	**PC WM**	**0.333**	**3.627**	**< 0.001**	**0.379**	**0.325**	**0.311**
			zAge	0.003	0.027	0.979	–0.221	0.003	0.002
			**zPTA4**	−**0.207**	−**20.80**	**0.040**	−**0.282**	−**0.194**	−**0.178**
dTOpt	0.172	7.69*	**PC WM**	**0.360**	**3.887**	**< 0.001**	**0.399**	**0.346**	**0.336**
			zAge	–0.063	–0.605	0.546	–0.229	–0.057	–0.052
			zPTA4	–0.074	–0.736	0.463	–0.188	–0.070	–0.064
dTOsyl	0.119	4.99**	**PC WM**	**0.222**	**2.324**	**0.022**	**0.290**	**0.215**	**0.207**
			zAge	–0.127	–1.178	0.241	–0.258	–0.111	–0.105
			zPTA4	–0.101	–0.971	0.334	–0.216	–0.092	–0.087
sylID	0.048	1.85	PC WM	0.219	2.042	0.043	0.259	0.167	0.164
			zAge	–0.029	–0.219	0.827	–0.202	–0.018	–0.018
			zPTA4	–0.041	–0.357	0.722	–0.174	–0.030	–0.029
TBAC6	0.282	14.52*	**PC WM**	**0.487**	**5.651**	**< 0.001**	**0.519**	**0.473**	**0.455**
			zAge	–0.027	–0.276	0.783	–0.251	–0.026	–0.022
			zPTA4	–0.099	–1.056	0.293	–0.223	–0.100	–0.085

Bold font highlights those independent variables having significant (p < 0.05) standardized Beta coefficients in significant regression solution. Asterisks mark significant F values for the regression solution: *p < 0.001; **p < 0.01; ***p < 0.05.

## Discussion

As was demonstrated in the top panel of Figure 2, the 34 YNH listeners in this study performed as expected, based on the normative data for the TBAC from Kidd et al. (2007). In addition, as had been found in Christopherson and Humes (1992) and Kidd et al. (2007), the TBAC scores were fairly reliable in older adults, although the reliability was enhanced considerably by averaging the scores for the 6 tonal auditory-discrimination tasks (TBAC6), as had been done by Humes et al. (2013b).

Differences in performance on the TBAC between the YNH and OHI groups, reported in the middle panel of Figure 2, were consistent with age-group differences reported previously by Humes and Christopherson (1991). Older adults performed significantly worse than young adults on several TBAC tests. Subsequent ANCOVA analyses with hearing loss (better-ear PTA4) and cognition (working memory, as indexed by PCwm) as covariates (Figure 2, bottom), however, suggested that the difference in TBAC scores between age groups was primarily due to group differences in working memory, rather than some unspecified age-related factor. Age-group effects disappeared when the covariates were used as statistical controls in ANCOVAs with working-memory performance being the lone significant factor in 7 of the 9 analyses, and one of two significant factors in one of the remaining two analyses. That is, working memory was a significant covariate for 8 of the 9 TBAC measures with age group being significant for only the temporal-order task using syllables. This is in line with the analyses for the TBAC described in Humes (1996) in which performance on the TBAC for two groups of adults differing in age but matched for hearing loss and cognition did not differ significantly.

The regression analyses summarized for all participants (Table 2) and for only the older adults (Table 3) provided further support for the predominant importance of working memory to TBAC performance across the adult lifespan. Significant regression solutions emerged for most TBAC measures in both sets of linear-regression analyses and the partial and part correlations supported the predominance of working memory in those analyses.

These findings should not be misunderstood as indicating older adults are expected to perform equivalently to young adults on the TBAC. Older adults performed worse than young adults on many of the TBAC tests (Figure 2, middle). Rather, this finding helps identify the factors underlying that observed age-group difference. It is the age-group difference in cognitive function, specifically working memory as measured here, that appears to underlie the poorer performance of older adults relative to young adults on the TBAC.

Although the focus here is on the TBAC, the link between auditory performance and working memory in older adults is not unique to the TBAC. Recently, Lentz, Humes and Kidd (in press), demonstrated similar links in this same study sample for over 20 psychoacoustic measurements spanning a much wider range of tasks than the TBAC. In those analyses, as was observed here, age alone seldom emerged as a significant predictor of psychoacoustic performance with working memory being the predominant predictor of performance. Unlike here, however, hearing loss was found to be a significant predictor on several psychoacoustic tasks as well, especially those tasks making use of stimuli that extended further into the high-frequency region of hearing loss than most stimuli in the TBAC.

Another important finding is that hearing loss, PTA4, was not related to TBAC performance for any of the tests, except for minor contributions to two TBAC measures within the group of 115 older adults (Table 3). The relative unimportance of hearing loss to TBAC performance had been noted previously (Humes and Christopherson, 1991) as a potential advantage in using the TBAC to assess auditory function in older adults, many of whom have significant hearing loss in the higher frequencies. Clearly, based on the audiograms in Figure 1, many of the older adults in this study had measurable hearing loss, especially in the higher frequencies. For the six tonal auditory-discrimination tasks in the TBAC, the stimuli are all in the mid-frequencies. Except for the embedded test-tone task, which makes use of frequencies varying between 300 and 3,000 Hz, the other five tone-based discrimination tasks in the TBAC use stimuli that are generally confined to 500–1,500 Hz which corresponds to the region of best hearing in older adults. Interestingly, among the older adults, the embedded test-tone task was one of two TBAC measures for which the better-ear PTA4 was found to be a significant secondary predictor (Table 3). To further minimize potential confounds of stimulus audibility in this study, a relatively high presentation level of 85 dB SPL was used for the TBAC. The absence of a significant effect of PTA4 on most of the TBAC tests further documents its utility as a measure of auditory function in older adults, including those with typical age-related hearing loss.

**FIGURE 3:**
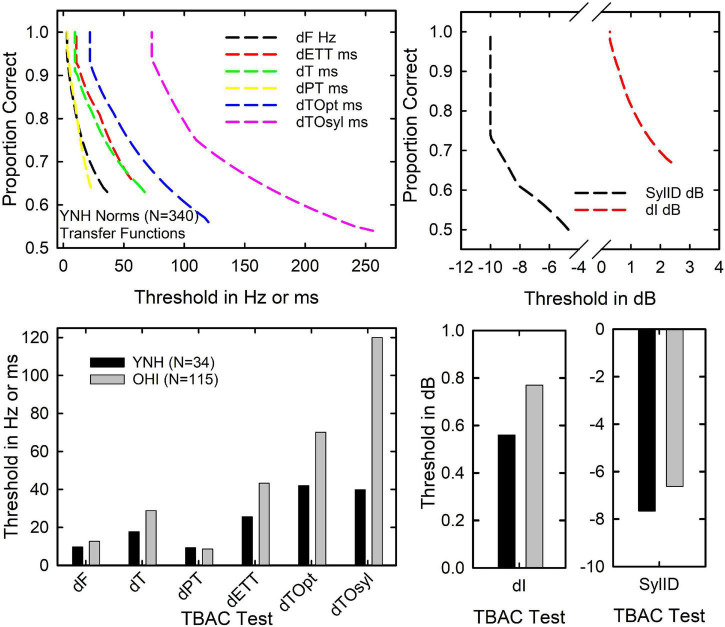
The top two panels show the transfer functions from the group data (*N* = 340) of Kidd et al. (2007) relating the proportion correct on a given TBAC test to the underlying stimulus dimension manipulated on that test. The lower two panels show the physical stimulus change needed at the median percent-correct performance on each TBAC test for the YNH (black bars) and OHI (gray bars) adults.

How do the age-group differences in percent-correct TBAC scores, such as those in the middle panel of Figure 2, translate to acoustical differences between the standard and comparison stimuli used in the various TBAC tests? To evaluate this, the median percent-correct scores for the YNH and OHI listeners were generated. Next, the group (*N* = 340) psychometric functions from Kidd et al. (2007), relating the proportion correct to the physical stimulus parameter manipulated on each TBAC test, were used as transfer functions to convert each median percent-correct score to a physical stimulus difference. The normative group psychometric functions for each of the nine TBAC tests are shown in the upper two panels of Figure 3. The median proportion-correct scores for the YNH and OHI groups were then converted to stimulus values in Hz, ms, or dB, depending on the test. The transformed medians appear as the black and gray vertical bars in the lower panels of Figure 3. For all TBAC tasks, lower values represent better performance. Except for the pulse-train (rhythm) discrimination task (dPT), the OHI listeners clearly required a larger difference between the standard and comparison stimuli at the median threshold for that group. The superior performance of the YNH group is probably most apparent for the two temporal-order tasks, dTOpt and dTOsyl, in the lower left panel. Here, on average, the OHI listeners required the durations of stimuli comprising a stimulus sequence to be 2–3 times longer (and the resulting rate of presentation to be slower) than that of the YNH group to discriminate between the standard and comparison sequences.

Fogerty et al. (2010) and Humes et al. (2010), in analyses of data from subject samples not overlapping with the present samples, reported age-group differences for temporal-order identification of brief vowel sequences. The temporal-order task was a monaural closed-set sequence-identification task rather than a diotic temporal-order discrimination task as in the dTOsyl test of the TBAC. Fogerty et al. (2010) included data from 35 young and 151 older adults for both a two-item and four-item vowel sequence. As in the present study, stimulus manipulations were applied to minimize the impact of age-group differences in hearing thresholds on temporal-order identification performance. Older adults were found to have significantly poorer temporal-order thresholds for both the two-vowel and four-vowel sequences. For the two-item temporal-order task, the median threshold of the older adults was more than three times greater than that of the younger adults. For the four-item task, the temporal-order threshold was 1.7 times longer than that for the young adults. Thus, the magnitude of this age-group difference in the thresholds for the temporal-order identification of syllable sequences in Fogerty et al. (2010) is comparable to the magnitudes of the differences shown for dTOsyl in Figure 3. Also consistent with the present findings for the TBAC in Table 3, Fogerty et al. (2010) found cognitive function to be the only significant predictor associated with individual differences in temporal-order identification within the group of older adults. Age-group differences in temporal-order identification performance were not examined with covariates by Fogerty et al. (2010) to determine the extent to which age-group differences in cognitive function may have mediated the observed age-group differences in temporal-order thresholds.

More recently, Humes et al. (2013a), again making use of a study sample independent of that in the present study, found temporal-order processing in hearing, vision, and touch to be strongly associated with cognitive function in a cross-sectional study of 245 young, middle-age, and older adults. Recently, in longitudinal follow-up analyses of 98 of the original 195 middle-age and older adults included in the cross-sectional study of Humes et al. (2013a), independent from this study sample, auditory temporal-order identification for brief syllables emerged as the most significant auditory measure in regression analyses predicting cognitive function, both for brief clinical cognitive assessments (Humes, 2020) and for comprehensive cognitive assessments (Humes, 2021). Both monaural and dichotic temporal-order identification measures were found to decline longitudinally, dichotic longitudinal declines also having been observed by Babkoff and Fostick (2017). Humes (2020, 2021) found both temporal-order identification measures to be predictive of declines in cognitive function in older adults over a 9-year period. Temporal-order processing typically explained 10–20% of the variance in cognitive function among middle-age and older adults using either form of cognitive assessment.

These prior and current findings reinforce the need to evaluate cognition when comparing the auditory performance of young and older adults as their declines in auditory abilities may be driven by differences in cognitive function. The focus in this study was on working memory and three different visual working-memory tasks were completed by all subjects. Although the tasks are considered working-memory measures, they may be considered relatively complex working-memory tasks compared to simpler measures such as forward or backward digit span. As a result of the complexity, other aspects of higher-level processing may be tapped beyond working memory alone. For example, one task required completion of a sequence of arithmetic operations, another required the reading and evaluation of sentences between items in the recall set (letters), and the third required spatial processing. The principal-components analysis was designed to capture the common working-memory component shared by all three tasks, thus providing a measure that excludes the task-specific variance. However, the task-specific abilities are also of interest because they are cognitive abilities that may be related to performance on the TBAC tasks, independent of the contribution of working memory. The relative strength of the correlations between specific working-memory tasks and TBAC performance among the older adults may vary across tasks, revealing selective influences of math, linguistic, and spatial abilities on the TBAC tasks. To evaluate this possibility, for the 115 older adults, correlations between the z-transformed TBAC and the three z-transformed working-memory scores were calculated and compared to that between the TBAC score and the overall working-memory principal component (the latter was not z-transformed because it already has a mean of 0 and standard deviation of 1 as a principal component score). Among the three working-memory tasks, performance on the sentence-span task had slightly but consistently higher correlations than the other two working-memory tasks across all TBAC measures. Moreover, the correlation between TBAC performance and performance on the sentence-span task was slightly but consistently higher than that for the overall working-memory principal-component. For example, averaged across all 8 TBAC scores and the TBAC6 average measure, 9 correlations in total, the mean correlation with the z-transformed sentence-span score was *r* = 0.42 versus *r* = 0.39 for the working-memory principal component. The difference between correlations was largest for the two speech-based TBAC tests, dTOsyl and sylID, and for two tasks with longer, more complex, sound sequences, dPT and dETT. For these four TBAC tests, the correlations with sentence-span score were 0.06–0.11 higher than those with the working-memory principal component (PCwm). The higher correlations for TBAC scores with sentence-span scores versus the overall working-memory principal component are relatively slight improvements. Nonetheless, these differences suggest that a working-memory measure involving the linguistic processing of visual stimuli correlates a bit more strongly with performance on the TBAC than a spatially based, arithmetic-based, or overall (PCwm) measure of working memory.

The mechanisms that underlie the observed correlations between performance on the TBAC and measures of working memory may also be related to task-specific aspects of the TBAC measures. That is, the implementation of the standard-two-alternative stimulus presentation format of the TBAC shares some characteristics of many working memory tasks. For all seven auditory-discrimination tasks, the standard stimulus is always presented first and then two additional stimuli are presented sequentially after that standard with only one differing from the standard. The task is to select the stimulus that differed from the standard. To do so, one must hold the standard in memory while performing comparisons with two subsequent stimuli. Further, an individual trial has sound durations for the standard and comparison stimuli that vary from task to task, with the longest stimuli occurring in the dPT, dETT, dTOpt, and dTOsyl tasks. Thus, although working memory is involved in the specific psychophysical procedure used in all TBAC tests, working memory may be taxed to a greater extent for those tasks with longer standard and comparison stimuli.

On the other hand, the concomitant decline in auditory abilities and cognition among older adults may offer insights into the factors underlying the well-established cognitive declines as adults advance in age (Humes et al., 2013a; Humes, 2020, 2021). That is, there may be shared underlying mechanisms that negatively impact both sensory and cognitive processing with increasing age either concomitantly or sequentially (Humes and Young, 2016).

Finally, as was noted previously, explaining the underlying mechanisms responsible for age-group differences in auditory abilities does not mean that older adults have auditory processing typical of that found in young adults. Older adults have difficulty processing many aspects of auditory stimuli. For sound sequences, older adults have considerable difficulty with rapid sequences (Figure 3). Knowing that this may be driven by underlying deficits in cognitive function does not change the fact that older adults have more difficulty processing fast sound sequences, it just explains why that difficulty is observed. Of course, running speech is a rapid sequence of sounds and the observed age-related deficit in temporal-order processing may underlie some of the speech-recognition difficulties experienced by older adults. However, temporal-processing measures were not significant predictors of aided speech understanding in Humes et al. (2013b) and were largely independent of speech measures in a study of auditory abilities in young listeners, using an expanded version of the TBAC (Kidd et al., 2007). The auditory task that accounted for most of the variance in speech understanding in both of those studies was the recognition (in noise) of familiar non-speech sounds. It may be that the cognitive changes that are associated with reduced temporal-processing abilities also have a negative impact on the recognition of spectrally and temporally complex familiar sounds, beyond their influence on the temporal-order TBAC measures.

## Data Availability Statement

The raw data supporting the conclusions of this article will be made available by the authors, without undue reservation.

## Ethics Statement

The studies involving human participants were reviewed and approved by IU Bloomington IRB. The patients/participants provided their written informed consent to participate in this study.

## Author Contributions

LH oversaw data analyses and was primarily responsible for the preparation of the manuscript. All authors shared responsibility equally for the design of the study, contributed to the writing of the manuscript, and approved the submitted version.

## Conflict of Interest

The authors declare that the research was conducted in the absence of any commercial or financial relationships that could be construed as a potential conflict of interest.

## Publisher’s Note

All claims expressed in this article are solely those of the authors and do not necessarily represent those of their affiliated organizations, or those of the publisher, the editors and the reviewers. Any product that may be evaluated in this article, or claim that may be made by its manufacturer, is not guaranteed or endorsed by the publisher.

## Footnotes

1
http://comdistec.com/new/TBAC.html

## References

[B1] ANSI (2004). *S3.6-2004, Specification for Audiometers.* New York, NY: American National Standards Institute.

[B2] Babkoff H. Fostick L. (2017). Age-related changes in auditory processing and speech perception: cross-sectional and longitudinal analyses. *Euro. J. Ageing* 14 269–281. 10.1007/s10433-017-0410-y 28936137 PMC5587455

[B3] Christopherson L. A. Humes L. E. (1992). Some psychometric properties of the Test of Basic Auditory Capabilities (TBAC). *J. Speech Hear. Res.* 35 929–935. 10.1044/jshr.3504.929 1405548

[B4] Cohen J. (1988). *Statistical Power Analysis for the Behavioral Sciences.* US: Lawrence Erlbaum Associates.

[B5] Fogerty D. Humes L. E. Kewley-Port D. (2010). Auditory temporalorder processing of vowel sequences by young and older adults. *J. Acoust. Soc. Am.* 127 2509–2520. 20370033 10.1121/1.3316291 PMC2865703

[B6] Folstein M. F. Folstein S. E. McHugh P. R. (1975). Mini-Mental State: a practical method for grading the cognitive state of patients for the clinician. *J. Psychiatr. Res.* 12 189–198. 1202204 10.1016/0022-3956(75)90026-6

[B7] Gallun F. J. Best V. (2020). “Age-related changes in segregation of sound sources,” in *Springer Handbook of Auditory Research, Aging and Hearing-Causes and Consequences*, ed. Helfer K. (Switzerland: Springer).

[B8] Hofer S. M. Sliwinski M. J. (2001). Understanding Ageing. An evaluation of research designs for assessing the interdependence of ageing-related changes. *Gerontology* 47 341–352. 10.1159/000052825 11721149

[B9] Humes L. E. (1996). Speech understanding in the elderly. *J. Am. Acad. Audiol.* 7 161–167. 8780988

[B10] Humes L. E. (2020). Associations between measures of auditory function and brief assessments of cognition. *Am. J. Audiol.* 29 825–837. 10.1044/2020_AJA-20-00077 32976027 PMC8608158

[B11] Humes L. E. (2021). Longitudinal changes in auditory and cognitive function in middle-aged and older adults. *J. Speech Lang. Hear. Res.* 64 230–249. 10.1044/2020_JSLHR-20-00274 33400551 PMC8608226

[B12] Humes L. E. Christopherson L. (1991). Speech-identification difficulties of the hearing-impaired elderly: the contributions of auditory-processing deficits. *J. Speech Hear. Res.* 34 686–693. 10.1044/jshr.3403.686 2072694

[B13] Humes L. E. Young L. A. (2016). Sensory-cognitive interactions in older adults. *Ear Hear.* 37 52S–61S. 10.1097/AUD.0000000000000303 27355770 PMC4930008

[B14] Humes L. E. Kidd G. R. Lentz J. J. (2013b). Auditory and cognitive factors underlying individual differences in aided speech-understanding among older adults. *Front. Syst. Neurosci.* 7:55. 10.3389/fnsys.2013.00055 PMC3787592 24098273

[B15] Humes L. E. Busey T. A. Craig J. Kewley-Port D. (2013a). Are age-related changes in cognitive function driven by age-related changes in sensory processing? *Atten. Percept. Psychophys.* 75 508–524. 10.3758/s13414-012-0406-9 23254452 PMC3617348

[B16] Humes L. E. Dubno J. R. Gordon-Salant S. Lister J. J. Cacace A. T. Cruickshanks K. J. (2012). Central presbycusis: a review and evaluation of the evidence. *J. Am. Acad. Audiol.* 23 635–666. 10.3766/jaaa.23.8.5 22967738 PMC5898229

[B17] Humes L. E. Kewley-Port D. Fogerty D. Kinney D. (2010). Measures of hearing threshold and temporal processing across the adult lifespan. *Hear. Res.* 264 30–40. 10.1016/j.heares.2009.09.010 19786083 PMC3182849

[B18] Humes L. E. Watson B. U. Christensen L. A. Cokely C. A. Halling D. A. Lee L. (1994). Factors associated with individual differences in clinical measures of speech recognition among the elderly. *J. Speech Hear. Res.* 37 465–474. 10.1044/jshr.3702.465 8028328

[B19] Kidd G. R. Watson C. S. Gygi B. (2007). Individual differences in auditory abilities. *J. Acoust. Soc. Am.* 122 418–435. 10.1121/1.2743154 17614500

[B20] Lewandowsky S. Oberauer K. Yang L.-X. Ecker U. K. H. (2010). A working memory test battery for MATLAB. *Behav. Res. Methods* 42 571–585. 10.3758/BRM.42.2.571 20479189

[B21] Salthouse T. A. (2010). *Major Issues in Cognitive Aging.* New York, NY: Oxford University Press.

[B22] Shrivastav M. N. Humes L. E. Aylsworth L. (2008). Temporal-order discrimination of tonal sequences by younger and older adults: the role of duration and rate. *J. Acoust. Soc. Am.* 124 462–471. 10.1121/1.2932089 18646990 PMC2809698

[B23] Studebaker G. A. (1985). A “rationalized” arcsine transform. *J. Speech Hear. Res.* 28, 455–462. 10.1044/jshr.2803.455 4046587

[B24] Surprenant A. M. Watson C. S. (2001). Individual differences in the processing of speech and nonspeech sounds by normal-hearing listeners. *J. Acoust. Soc. Am.* 110 2085–2095. 10.1121/1.1404973 11681386

[B25] Watson B. U. Miller T. K. (1993). Auditory perception, phonological processing and reading ability/disability. *J. Speech Hear. Res.* 36 850–863. 10.1044/jshr.3604.850 8377497

[B26] Watson C. S. (1987). “Uncertainty, informational masking, and the capacity of immediate auditory memory,” in *Auditory Processing of Complex Sounds*, eds Yost W. A. Watson C. S. (Hillsdale, NJ: Lawrence Erlbaum), 267–277.

[B27] Watson C. S. Jensen J. K. Foyle D. C. Leek M. R. Goldgar D. E. (1982a). Performance of 146 normal adult listeners on a battery of auditory discrimination tasks. *J. Acoust. Soc. Am.* 71:S73. 10.1121/1.5134059

[B28] Watson C. S. Johnson D. M. Lehman J. R. Kelly W. J. Jensen J. K. (1982b). An auditory discrimination test battery. *J. Acoust. Soc. Am.* 71:S73.

[B29] Wechsler D. (1981). *The Wechsler Adult Intelligence Scale-Revised.* New York: The Psychological Corporation.

[B30] Wechsler D. (1983). *The Wechsler Memory Scale-Revised.* New York: The Psychological Corporation.

[B31] World Health Organization (2001). *International Classification of Functioning, Disability and Health.* Geneva: World Health Organization.

[B32] World Health Organization (2021). *World Report on Hearing.* Geneva: World Health Organization.

